# Skin autofluorescence, a non-invasive biomarker for advanced glycation end products, is associated with the metabolic syndrome and its individual components

**DOI:** 10.1186/s13098-017-0241-1

**Published:** 2017-05-30

**Authors:** Robert P. van Waateringe, Sandra N. Slagter, Andre P. van Beek, Melanie M. van der Klauw, Jana V. van Vliet-Ostaptchouk, Reindert Graaff, Andrew D. Paterson, Helen L. Lutgers, Bruce H. R. Wolffenbuttel

**Affiliations:** 1 Department of Endocrinology, University of Groningen, University Medical Center Groningen, HPC AA31, P.O. Box 30001, 9700 RB Groningen, The Netherlands; 2 Program in Genetics and Genome Biology, Hospital for Sick Children, Toronto, ON M5G 0A4 Canada; 3 Department of Internal Medicine, Medical Center Leeuwarden, 8934 AD Leeuwarden, The Netherlands

## Abstract

**Background:**

The metabolic syndrome (MetS) comprises several cardiometabolic risk factors associated with increased risk for both type 2 diabetes and cardiovascular disease. Skin autofluorescence (SAF), a non-invasive biomarker of advanced glycation end products accumulation, is associated with cardiovascular complications in subjects with diabetes. The aim of the present study was to examine the association between SAF and the presence of MetS as well as its individual components in a general population.

**Methods:**

For this cross-sectional analysis, we included 78,671 non-diabetic subjects between 18 and 80 years of age who participated in the LifeLines Cohort Study and had SAF measurement obtained non-invasively using the AGE Reader. MetS was defined according to the revised NCEP ATP III criteria. Students unpaired *t* test was used to test differences between groups. Both logistic and linear regression analyses were performed in order to test associations between the individual MetS components and SAF.

**Results:**

Subjects with MetS had higher SAF (2.07 ± 0.45 arbitrary units, AU) compared to individuals without MetS (1.89 ± 0.42 AU) (p < 0.001). There was a positive association between the number of MetS components and higher SAF Z-scores (p < 0.001). Individuals in the highest SAF tertile had a higher presence of MetS (OR 2.61; 95% CI 2.48–2.75) and some of the individual components compared to subjects in the lowest SAF tertile. After correction for age, gender, creatinine clearance, HbA1c and smoking status, only elevated blood pressure and low HDL cholesterol remained significantly associated with higher SAF (p = 0.002 and p = 0.001 respectively).

**Conclusion:**

Skin autofluorescence was associated with the presence of MetS and some of its individual components. In addition, increasing SAF Z-scores were observed with a higher number of MetS components. Prospective studies are needed to establish whether SAF can be used as an (additional) screening tool to predict both cardiovascular disease and type 2 diabetes in high-risk populations.

## Background

Advanced glycation end products (AGEs) comprise a group of largely irreversibly glycated proteins, lipids and nucleic acids which represent chronic exposure to hyperglycaemia and oxidative stress [1, 2]. AGEs are formed via several pathways, usually the Maillard reaction between carbonyl groups of reducing sugars and free amino groups from proteins. AGEs accumulate in the skin as a result of ageing [3, 4]. The formation and accumulation may be increased as a result of both endogenous and exogenous factors, including hyperglycaemia in diabetes, impaired renal excretion in subjects with kidney failure, as well as dietary intake and tobacco smoking [5–8].

Since the past decade, it has become possible to estimate tissue AGE accumulation non-invasively by measuring autofluorescence of the skin (SAF) [9]. In our previous study we showed that SAF was associated with several clinical and lifestyle parameters [10]. SAF has previously been validated against tissue AGE measurements and reference values against age were obtained [11, 12]. SAF was reported to be elevated in subjects with type 1 and 2 diabetes [9, 13]. Moreover, SAF has been shown to be a strong predictor of long-term cardiovascular complications and mortality in both type 1 and type 2 diabetes and end-stage renal failure [14–18]. In addition, recent studies have shown higher SAF levels to be associated with coronary artery disease, peripheral artery disease and (sub)clinical atherosclerosis independent of diabetes [19–21].

The metabolic syndrome (MetS) is a cluster of cardiometabolic abnormalities associated with increased risk for cardiovascular disease (CVD) and type 2 diabetes mellitus [22, 23]. The MetS is a worldwide problem which prevalence increases worldwide, particularly due to the growing epidemic of obesity [24–26]. Glycation is known to play an essential role in the mechanism that leads to the formation of AGEs [6].

It has been shown that AGEs increase inflammation and oxidative stress hereby promoting insulin resistance. On the other hand, a low AGEs diet improves insulin sensitivity [27, 28].

However, the association of the other cardiometabolic components with SAF has not been assessed in detail. As SAF measurement might be used as a future screening tool in high-risk populations, such as the MetS, to refine estimation of risk of future cardiovascular events or development of type 2 diabetes, knowledge of potential associations with SAF is important. Therefore, the aim of this cross-sectional study was to assess SAF in subjects with MetS. We examined the association between the individual MetS components and SAF in a large-scale general population.

## Methods

### Participants

Subjects included were participants from the LifeLines Cohort Study [29], a large population-based cohort study in the northern region of the Netherlands examining the interaction between genetic and environmental factors associated with chronic diseases and healthy ageing. Between 2006 and 2013, individuals from the three northern provinces of the Netherlands were invited to participate in the study. At baseline, both physical examination and extensive questionnaires were collected from more than 167,000 participants [30]. All participants have provided written informed consent before participating in the study. The study has been approved by the Medical Ethics Review Committee of the University Medical Center Groningen. For the present study, we included subjects of Western European descent between 18 and 80 years of age having a SAF measurement available (n = 82,515). We excluded subjects with either missing data for MetS status (n = 1221) and those with a serum creatinine >140 µmol/L (n = 75), as severely reduced kidney function itself increases AGE levels. Furthermore, subjects with missing data for diabetes status (n = 86) were excluded, as were those with type 1 diabetes (n = 177), type 2 diabetes (n = 2157) or previous gestational diabetes (n = 128) leaving 78,671 non-diabetic individuals for analyses.

### Clinical and lifestyle data

The following clinical data were used: age, gender, body mass index (BMI), waist circumference, systolic and diastolic blood pressure, serum lipids, fasting plasma glucose, HbA1c, creatinine clearance, use of medication, and self-reported history of CVD (myocardial infarction and cerebrovascular accident). Information regarding smoking behaviour was collected by extensive questionnaires, as described earlier [10]. Pack-years of smoking were calculated as the number of cigarette packages smoked per day multiplied by the number of years an individual had smoked. Data regarding coffee consumption (cups of coffee per day) were obtained by questionnaire. We were not able to distinguish between caffeinated and decaffeinated coffee consumption.

### Physical measurements

Weight was measured to the nearest 0.1 kg and height and waist circumference to the nearest 0.5 cm by trained technicians using calibrated measuring equipment, with participants wearing light clothing and no shoes. Waist circumference was measured with a tape around the body between the lower rib margin and the iliac crest. BMI was calculated as weight divided by height squared (kg/m^2^). Systolic and diastolic blood pressure were measured every minute during 10 min in the supine position using an automated Dinamap Monitor (GE Healthcare, Freiburg, Germany). The average of the last three readings was recorded. SAF was assessed as the mean of three consecutive measurements using the AGE Reader (DiagnOptics Technologies BV, Groningen, the Netherlands) in all participants, as described previously [9–11].

### Biochemical measures

Blood samples were taken in the fasting state between 8.00 and 10.00 a.m. and transported to the LifeLines laboratory facility at room temperature or at 4 °C, depending on the sample requirements. On the day of collection, HbA1c (EDTA-anticoagulated) was analyzed using a NGSP-certified turbidimetric inhibition immunoassay on a Cobas Integra 800 CTS analyzer (Roche Diagnostics Nederland BV, Almere, the Netherlands). Serum creatinine was measured on a Roche Modular P chemistry analyzer (Roche, Basel Switzerland), and creatinine clearance was calculated with the chronic kidney disease epidemiology collaboration (CKD-EPI) formula [31]. Total and high density lipoprotein (HDL) cholesterol were measured using an enzymatic colorimetric method, triglycerides using a colorimetric UV method, and low density lipoprotein (LDL) cholesterol using an enzymatic method, on a Roche Modular P chemistry analyzer (Roche, Basel, Switzerland). Fasting blood glucose was measured using a hexokinase method.

### Definition of the metabolic syndrome

Diagnosis of MetS was established if a subject satisfied at least three out of five criteria according to the revised National Cholesterol Education Programs Adults Treatment Panel III (NCEP ATPIII criteria) [22]: (1) systolic blood pressure ≥130 mmHg and/or diastolic blood pressure ≥85 mmHg and/or use of antihypertensive medication; (2) HDL cholesterol levels <1.03 mmol/L in men and <1.30 mmol/L in women and/or use of lipid-lowering medication influencing HDL levels; (3) triglyceride levels ≥1.70 mmol/L and/or use of triglyceride-lowering medication; (4) waist circumference ≥102 cm in men and ≥88 cm in women; (5) fasting glucose level between 5.6 and 7.0 mmol/L. As mentioned earlier, people with diabetes were excluded from analysis to reflect ‘true’ MetS.

### Calculations and statistical analyses

Data are shown as mean ± standard deviation (SD) or median and interquartile range (IQR) in case of non-normally distributed data. Student’s unpaired *t* test, Analysis of Variance (ANOVA) or Chi Square test were performed to compare groups. Age-adjusted SAF levels (Z-scores) were calculated based on the total population (separated for males and females) as SAF and MetS are strongly affect by ageing [10, 32]. Next, we classified individuals into tertiles of their age-adjusted SAF Z-scores, in men: lowest SAF Z ≤ −0.59, intermediate SAF −0.60 < Z < 0.49 and highest SAF Z ≥ 0.50; in women: lowest SAF Z ≤ −0.72, intermediate SAF −0.73 < Z < 0.33 and highest SAF Z ≥ 0.34. Logistic regression analysis was performed to assess whether either of the SAF groups (low SAF was set as a reference) were associated with the presence of MetS and its components. Model 1 shows the unadjusted association between tertiles of SAF Z-scores and MetS as well as its individual components. In model 2, we adjusted for gender and BMI. In model 3, we additionally adjusted for creatinine clearance, HbA1c, current smoking, pack-years and CVD history. Linear regression analysis was performed to examine the association between each of the individual MetS components and SAF among the population with MetS. In the multivariate models, we adjusted for determinants of SAF reported in our previous study, including age, gender, BMI, creatinine clearance, HbA1c, smoking status, packyears, and CVD history [10]. SPSS (version 22, IBM, Armonk, NY, USA) was used for statistical analyses. A *p* value <0.001 (two-tailed) was considered statistically significant.

## Results

### Clinical characteristics

Table 1 shows the clinical characteristics of the study population stratified for gender and MetS status. The overall prevalence of MetS was 19% in men and 12% in women.

**Table 1:** Clinical characteristics of the study population stratified by MetS status and gender

Characteristic	Men (n = 32,601)		Women (n = 46,070)	
	MetS	Without MetS	MetS	Without MetS
n (%)	6220 (19)	26,381 (81)	5367 (12)	40,703 (88)
Age (years)	49 ± 11*	44 ± 13	50 ± 12*	44 ± 12
Body mass index (kg/m^2^)	29.8 ± 3.6*	25.5 ± 3.1	30.6 ± 5.1*	25.1 ± 4.1
Waist circumference (cm)	106 ± 9*	93 ± 9	100 ± 11*	85 ± 11
Systolic blood pressure (mmHg)	141 ± 14*	129 ± 14	138 ± 16*	121 ± 15
Diastolic blood pressure (mmHg)	82 ± 9*	76 ± 9	79 ± 9*	72 ± 9
Total cholesterol (mmol/L)	5.4 ± 1.1*	5.1 ± 1.0	5.4 ± 1.1*	5.0 ± 1.0
HDL cholesterol (mmol/L)	1.04 ± 0.23*	1.37 ± 0.30	1.24 ± 0.30*	1.65 ± 0.38
LDL cholesterol (mmol/L)	3.56 ± 0.93*	3.35 ± 0.89	3.57 ± 0.95*	3.05 ± 0.88
Triglycerides (mmol/L)	2.03 (1.60–2.73)*	1.04 (0.77–1.40)	1.72 (1.18–2.14)*	0.83 (0.64–1.11)
Creatinine clearance (mL/min)^a^	137 ± 36*	124 ± 28	125 ± 39*	111 ± 29
Creatinine clearance (mL/min)^b^	130 ± 18*	128 ± 16	135 ± 21	134 ± 18
Fasting plasma glucose (mmol/L)	5.45 ± 0.55*	4.98 ± 0.43	5.33 ± 0.61*	4.75 ± 0.42
HbA1c (%)	5.7 ± 0.3*	5.5 ± 0.3	5.7 ± 0.4*	5.5 ± 0.3
HbA1c (mmol/mol)	38.5 ± 3.8	36.7 ± 3.2	39.3 ± 3.9	36.5 ± 3.2
Statin use, n (%)	783 (13)*	1260 (5)	554 (10)*	1029 (3)
TG-lowering medication, n (%)	44 (1)*	9 (0.01)	16 (0.3)*	3 (0.01)
BP-lowering medication, n (%)	1445 (23)*	2008 (8)	1919 (36)*	3099 (8)
MI, n (%)	214 (3.5)*	289 (1.1)	56 (1.0)*	94 (0.2)
CVA, n (%)	70 (1.1)*	188 (0.7)	66 (1.2)*	203 (0.5)
Coffee consumption (cups per day)	4.7 (2.8–5.6)*	3.7 (2.8–5.6)	3.3 (1.9–4.7)*	2.8 (1.3–4.7)
Smoking status, n (%)				
Non-smokers	2117 (34)*	12,259 (47)	2203 (41)*	19,836 (49)
Ex-smokers	2425 (39)	8271 (32)	1899 (36)	12,847 (32)
Current smokers	1629 (27)	5677 (21)	1228 (23)	7698 (19)
Pack-years				
Ex-smokers	12.8 (6.5–21.9)*	8.2 (3.8–15.0)	7.8 (3.5–15.3)*	5.2 (2.2–10.4)
Current-smokers	18.0 (10.7–27.0)*	12.9 (6.4–21.5)	16.9 (9.6–26.3)*	11.2 (5.4–18.8)
SAF (AU)	2.07 ± 0.44*	1.94 ± 0.43	2.07 ± 0.45*	1.86 ± 0.42
SAF Z-score	0.21 ± 0.02*	0.04 ± 0.01	0.19 ± 0.02*	−0.10 ± 0.01

Data are presented as mean ± standard deviation, or median (interquartile range) and number (%)

*AU* arbitrary units, *BP* blood pressure, *CVA* cerebrovascular accident, *GFR* Glomerular Filtration Rate, *HDL* high density lipoprotein, *LDL* low density lipoprotein, *MetS* metabolic syndrome, *Mi* myocardial infarction, *SAF* skin autofluorescence, *TG* triglycerides

* p value <0.001

^a^Creatinine clearance (Cockcroft-Gault formula), ^b^ Creatinine clearance (CKD EPI)

In both genders, individuals with MetS were older than subjects without MetS and had a significantly higher BMI and waist circumference (all p < 0.001). Both in men and women, the prevalence of MI (3.5% in men and 1.0% in women) and CVA (1.1% in men and 1.2% in women) was higher among individuals with MetS compared to subjects without MetS (p < 0.001). The percentage of current smokers as well as the number of pack-years smoked (p < 0.001) were higher in the participants with MetS. In addition, both men and women with MetS reported higher consumption of coffee compared to individuals without MetS (p < 0.001). Mean SAF levels were significantly higher among both male and female subjects with MetS compared to individuals without MetS (men: 2.07 ± 0.44 AU vs 1.94 ± 0.43 AU, p < 0.001; women: 2.07 ± 0.45 AU vs 1.86 ± 0.42 AU, p < 0.001). In subjects with MetS, mean SAF levels were not different between men and women (2.07 ± 0.44 AU vs 2.07 ± 0.45 AU, p = 0.715). However, among individuals without MetS, men had significantly higher SAF levels than women (1.94 ± 0.43 AU vs 1.86 ± 0.42 AU, p < 0.001) (data not shown). Furthermore, mean SAF was 2.33 ± 0.50 AU in subjects with a history of MI and 1.91 ± 0.43 AU in subjects without a history of MI (p < 0.001). Mean SAF was 2.22 ± 0.50 AU in subjects with a history of CVA and 1.91 ± 0.43 AU in subjects without a history of CVA (p < 0.001) (data not shown). Finally, within obese individuals, subjects with MetS had a higher SAF than individuals without MetS (2.11 ± 0.47 AU vs 1.94 v 0.41 AU, p < 0.001). However, within the population with MetS, there was no significant difference in SAF between obese and non-obese individuals (2.07 ± 0.45 AU vs 2.06 v 0.45 AU, p = 0.46) (data not shown).

### The association between SAF and the number of MetS components

**Fig. 1:**
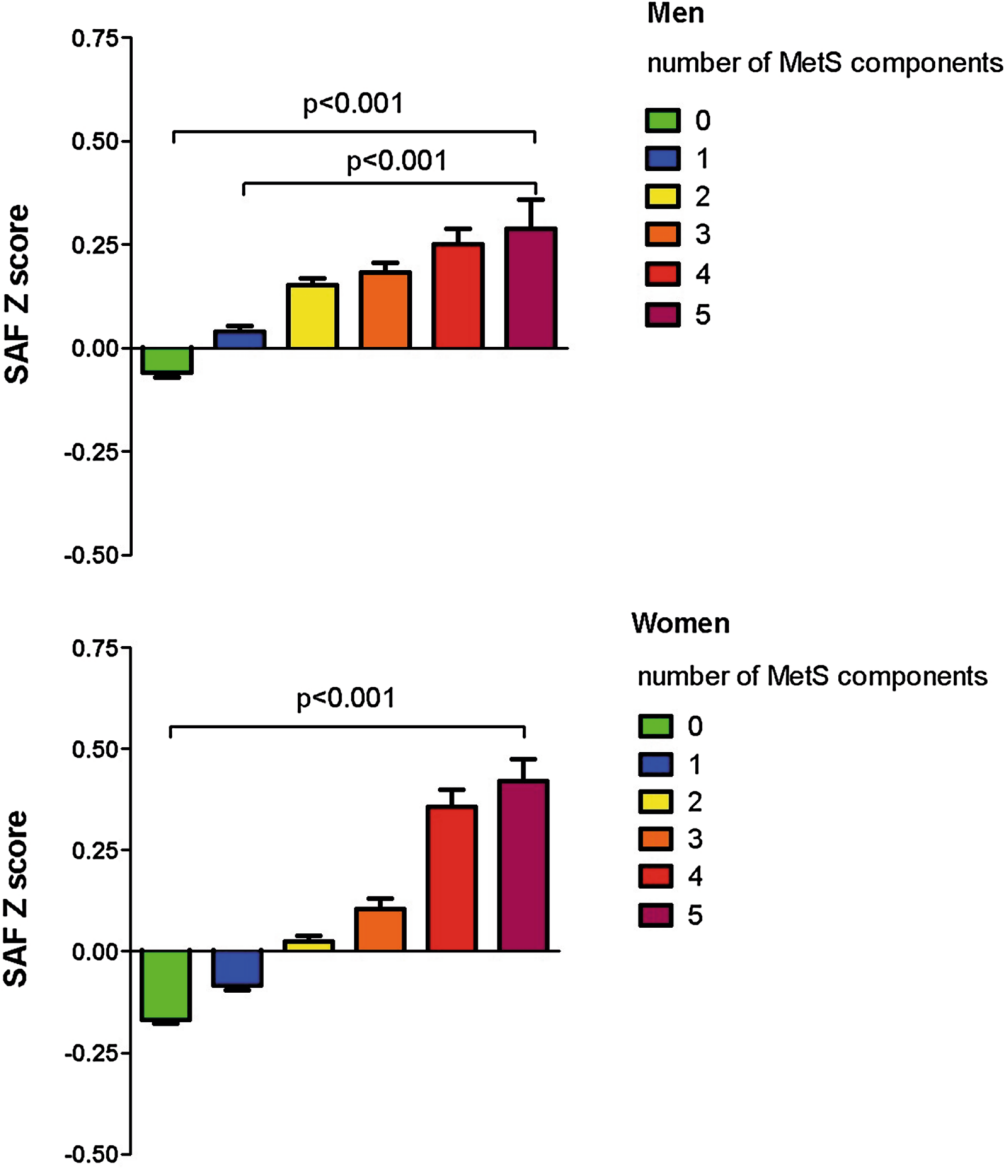
Age-adjusted SAF Z-scores increase with a higher number of MetS components. *Bars* reflect mean SAF Z-scores ± standard error. Men: no MetS component (n = 8999), one MetS component (n = 10,535), two MetS components (n = 6847), three MetS components (3955), four MetS components (n = 1821), five MetS components (n = 445). Women: no MetS component (n = 17,062), one MetS component (n = 14,423), two MetS components (n = 928), three MetS components (n = 3725), four MetS components (n = 1365), five MetS components (n = 277). *SAF* skin autofluorescence, *MetS* metabolic syndrome

In both men and women, we observed a gradual rise in age-adjusted SAF Z-scores with higher number of individuals MetS components. Compared to men without any MetS components, SAF Z-scores were significantly higher among subjects with ≥1 MetS component (p < 0.001). Men with ≥2 MetS components had significantly higher SAF Z-scores compared to men having only one MetS component (p < 0.001). No significant differences in SAF Z-scores were observed between men with two vs three MetS components as well as men with four vs five MetS components. Among women, SAF Z-scores were significantly higher among those with ≥1 MetS component compared to women without any MetS components (p < 0.001). Women with three, four or five MetS components had significantly higher SAF Z-scores compared to women with only one MetS component (p < 0.001). No differences in SAF Z-scores were found for women with two vs one MetS components as well as three vs two MetS components. Furthermore, women with either four or five MetS components had significantly higher SAF Z-scores than women with two MetS components or three MetS components (p < 0.001). In men, SAF Z-scores tended to be higher in those with one, two and three MetS components while in women SAF Z-scores were higher in three, four or five MetS components compared to men (Fig. 1).

### SAF and the prevalence of the individual MetS components

**Fig. 2:**
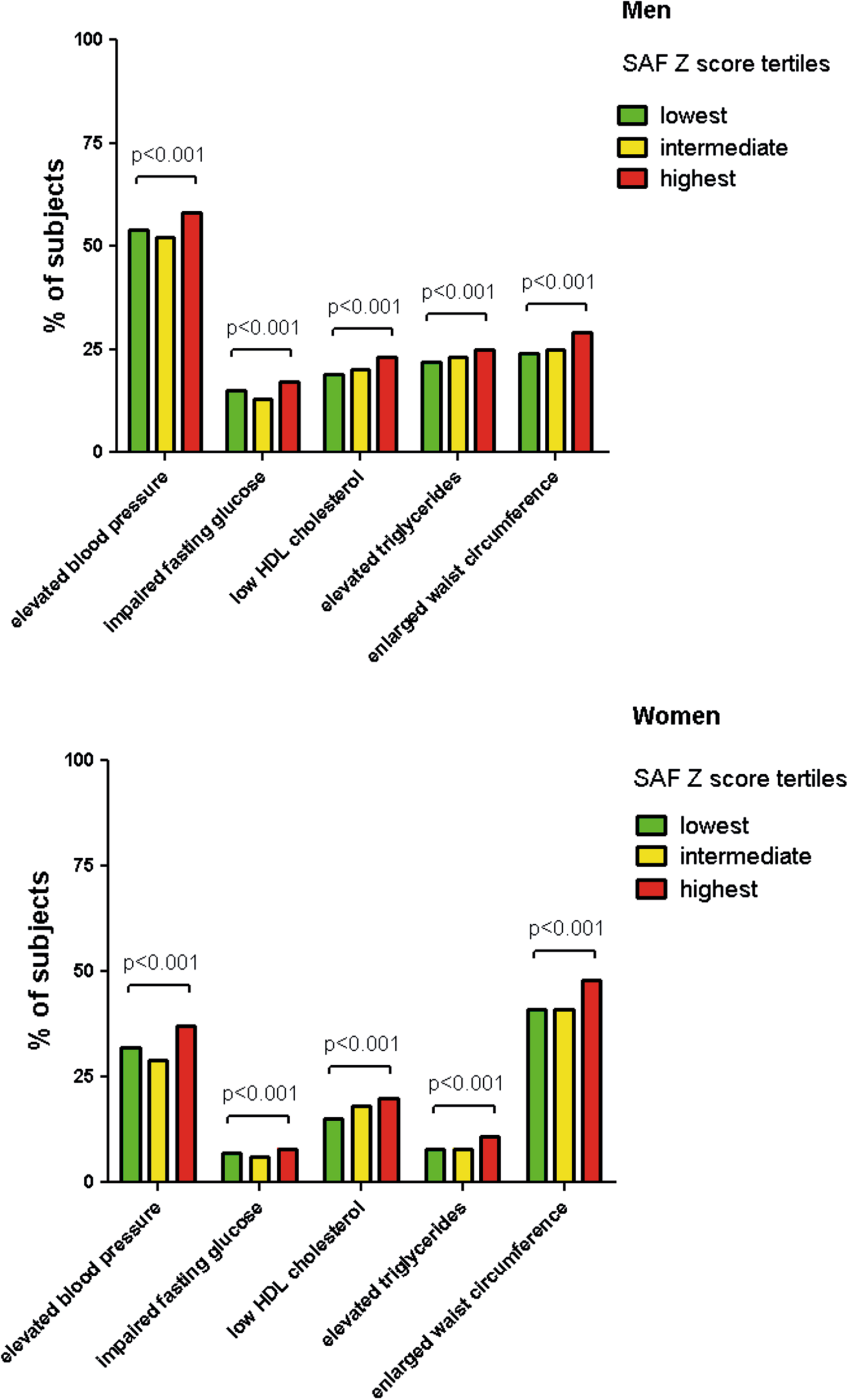
Prevalence of the individual MetS components according to tertiles of age-adjusted SAF Z-scores. *Bars* show percentage of the metabolic syndrome components. Lowest, intermediate and highest group are based on tertiles of age-adjusted SAF Z-scores. *SAF* skin autofluorescence

Figure 2 shows the prevalence of the individual MetS components for both men and women in the total study population according to tertiles of age-adjusted SAF Z-scores. Among men, individuals in the highest SAF groups had a higher prevalence of elevated blood pressure (58%) compared to subjects in the middle (52%) and lowest SAF group (54%).

We observed the same trend for enlarged waist circumference, impaired fasting glucose, elevated triglycerides and low HDL cholesterol (all p < 0.001).

In contrast to men, the most frequent MetS component in women was enlarged waist circumference (high SAF 48%, intermediate SAF 41% and lowest SAF 41%). There was a clear and significant trend among women within the highest SAF group, to be associated with higher prevalence of blood pressure, elevated triglycerides, lower HDL cholesterol levels and impaired fasting glucose (all p < 0.001).

### Logistic regression analysis for the presence of MetS and it components across SAF tertiles

Table 2 shows the results of the logistic regression analyses describing the association between SAF tertiles and the presence of MetS in the total population. Compared to the low SAF group which was set as a reference, high SAF was significantly associated with a higher presence of MetS (odds ratio (OR) 2.61; 95% CI 2.48–2.75). After adjusting for age, gender, BMI, creatinine clearance, HbA1c, smoking status, packyears, CVD history, high SAF (OR 1.24; 95% CI 1.13–1.37) and intermediate SAF (OR 1.11; 95% CI 1.01–1.22) remained significantly associated with the presence of MetS (Table 2, model 3). Considering the individual MetS components, high SAF (OR 3.16; 95% CI 2.97–3.37 and intermediate SAF (OR 1.90; 95% CI 1.78–2.03) were significantly associated with the presence of impaired fasting glucose, although the OR attenuated after adjusting for several covariables (Table 2, models 1, 2 and 3). We also found high SAF (OR 2.93; 95% CI 2.82–3.03) and intermediate SAF (OR 1.71; 95% CI 1.65–1.77) to be associated with the presence of elevated blood pressure (Table 2, model 1). Finally, high SAF (OR 1.93; 95% CI 1.86–2.00) and intermediate SAF (OR 1.45; 95% CI 1.40–1.50) were also associated with the presence of enlarged waist circumference, which remained significant after adjusting for covariables (Table 2, models 1, 2 and 3).

**Table 2:** Unadjusted and adjusted odds ratios for metabolic syndrome and its components associated with tertiles of SAF (total population)

Metabolic syndrome	Model 1		Model 2		Model 3	
	OR	p value	OR	p value	OR	p value
SAF (continuous)	2.37 (2.27−2.48)	<*0.0001*	1.36 (1.28–1.44)	<*0.0001*	1.17 (1.08–1.26)	<*0.0001*
Low SAF	Ref.		Ref.		Ref.	
Intermediate SAF	1.71 (1.62–1.81)	<*0.0001*	1.13 (1.06–1.21)	<*0.0001*	1.11 (1.01–1.22)	*0.031*
High SAF	2.61 (2.48–2.75)	<*0.0001*	1.38 (1.29–1.48)	<*0.0001*	1.24 (1.13–1.37)	<*0.0001*
Elevated blood pressure						
SAF (continuous)	3.03 (2.92–3.14)	<*0.0001*	1.25 (1.20–1.31)	<*0.0001*	1.21 (1.14–1.29)	<*0.0001*
Low SAF	Ref.		Ref.		Ref.	
Intermediate SAF	1.71 (1.65–1.77)	<*0.0001*	1.06 (1.02–1.11)	*0.005*	1.04 (0.98–1.11)	0.220
High SAF	2.93 (2.82–3.03)	<*0.0001*	1.18 (1.12–1.24)	<*0.0001*	1.16 (1.08–1.24)	<*0.0001*
Impaired fasting glucose						
SAF (continuous)	2.59 (2.47–2.72)	<*0.0001*	1.17 (1.10–1.25)	<*0.0001*	1.09 (1.00–1.19)	*0.049*
Low SAF	Ref.		Ref.		Ref.	
Intermediate SAF	1.90 (1.78–2.03)	*<0.0001*	1.09 (1.01–1.17)	*0.026*	1.08 (0.97–1.20)	0.185
High SAF	3.16 (2.97–3.37)	*<0.0001*	1.34 (1.24–1.45)	*<0.0001*	1.27 (1.14–1.42)	*<0.0001*
Low HDL cholesterol						
SAF (continuous)	1.08 (1.03–1.12)	*0.001*	1.46 (1.39–1.54)	<*0.0001*	1.27 (1.19–1.36)	<*0.0001*
Low SAF	Ref.		Ref.		Ref.	
Intermediate SAF	0.96 (0.92–1.01)	0.082	1.08 (1.02–1.13)	0.004	1.02 (0.94–1.10)	0.663
High SAF	1.01 (0.96–1.05)	0.786	1.27 (1.20–1.34)	<*0.0001*	1.13 (1.04–1.22)	*0.003*
Elevated triglycerides						
SAF (continuous)	1.78 (1.70–1.85)	<*0.0001*	1.29 (1.22–1.37)	<*0.0001*	1.08 (1.00–1.16)	*0.029*
Low SAF	Ref.		Ref.		Ref.	
Intermediate SAF	1.49 (1.41–1.57)	<*0.0001*	1.12 (1.05–1.18)	<*0.0001*	1.04 (0.95–1.13)	0.426
High SAF	1.89 (1.80–1.98)	<*0.0001*	1.34 (1.25–1.42)	<*0.0001*	1.17 (1.07–1.28)	0.001
Enlarged waist circumference						
SAF (continuous)	1.89 (1.82–1.95)	<*0.0001*	1.31 (1.23–1.39)	<*0.0001*	1.32 (1.21–1.44)	<*0.0001*
Low SAF	Ref.		Ref.		Ref.	
Intermediate SAF	1.45 (1.40–1.50)	<*0.0001*	1.11 (1.05–1.18)	*0.001*	1.14 (1.04–1.25)	*0.004*
High SAF	1.93 (1.86–2.00)	<*0.0001*	1.26 (1.18–1.35)	<*0.0001*	1.32 (1.20–1.46)	<*0.0001*

Data are presented as odds ratios (95% confidence interval) per arbitrary unit (AU). SAF groups were based on tertiles of age-adjusted SAF Z-scores. Model 1 = unadjusted; model 2 = adjusted for age, gender, BMI; model 3 = adjusted for age, gender, BMI, HbA1c, creatinine clearance, current smoking, pack-years, MI and CVA

Significant associations are shown in italic

*BMI* body mass index, *CVA* cerebrovascular accident, *HDL* high density lipoprotein, *MI* myocardial infarction, *OR* odds ratio, *SAF* skin autofluorescence

### Associations for SAF in the metabolic syndrome population

The univariate associations between the individual MetS components and SAF levels in the MetS population are shown in Table 3. In subjects with MetS, the impaired fasting glucose and elevated blood pressure components gave the strongest increase in SAF. Subjects with MetS having the impaired fasting glucose component had a 0.12 AU higher SAF compared to subjects without this particular component. A similar association was observed for individuals with MetS having the elevated blood pressure who had a 0.11 AU higher SAF than those without elevated blood pressure. Multivariate analyses showed that after adjusting for several determinants of SAF, elevated blood pressure (0.05 AU) and low HDL cholesterol (0.04 AU) were significantly associated with higher SAF. Impaired fasting glucose, elevated triglycerides and enlarged waist circumference were not significantly associated with SAF. Regarding the clinical and lifestyle factors, age, HbA1c, current smoking, packyears and a history of CVD were all significantly associated with higher SAF whereas a higher creatinine clearance was associated with lower SAF.

**Table 3:** Associations for skin autofluorescence in the metabolic syndrome population

	Coefficient B	SE	p value
Univariate model			
Elevated blood pressure	0.113	0.012	<0.0001
Low HDL cholesterol	−0.094	0.009	<0.001
Elevated triglycerides	0.029	0.009	0.001
Impaired fasting glucose	0.124	0.008	<0.0001
Enlarged waist circumference	0.038	0.011	0.001
Multivariate model			
Elevated blood pressure	0.044	0.015	*0.003*
Low HDL cholesterol	0.036	0.012	*0.002*
Elevated triglycerides	−0.001	0.011	0.920
Impaired fasting glucose	0.008	0.012	0.502
Enlarged waist circumference	0.016	0.014	0.269
Age	0.019	0.001	*1.3* *×* *10* ^*−159*^
Male gender	−0.016	0.011	0.272
Creatinine clearance (mL/min)	0.001	0.0002	*0.014*
HbA1c (%)	0.055	0.016	*2.3* *×* *10* ^*−4*^
Coffee consumption (cups/day)	0.024	0.002	*6.6* *×* *10* ^*−29*^
Current smoking^a^	0.116	0.011	*1.1* *×* *10* ^*−23*^
Pack-years	0.004	0.0003	*5.6* *×* *10* ^*−31*^
MI	0.062	0.029	*0.033*
CVA	0.026	0.043	0.562

Data are shown as coefficient B (standard error) per arbitrary unit (AU). The individual MetS components were included into the model as categorical variables (yes/no) while the other variables were continuous variables

Significant associations are shown in italic

*CVA* cerebrovascular accident, *HDL* high density lipoprotein, *MI* myocardial infarction

^a^Current smoking vs non- and former smoking

## Discussion

In the present study, we have demonstrated that SAF levels were higher in both male and female participants with MetS compared to those without MetS. Furthermore, SAF was significantly and independently associated with the presence of MetS and some of its individual components, particularly elevated blood pressure, impaired fasting glucose and enlarged waist circumference.

One of the main findings of the present study was that we observed significantly higher SAF levels in subjects with MetS compared to individuals without MetS, which is in line with a study by Den Engelsen et al. [33]. These authors observed higher SAF levels in obese subjects with MetS compared to those without MetS. The latter group may be considered as “healthy obese”, which is also reflected by their SAF level. Additional analyses on our data showed a similar pattern (data not shown). In our study, 45% of men and 51% of women with MetS were obese, as defined by a BMI above 30 kg/m^2^. However, within the population with MetS, we did not observe a statistically significant difference in SAF between obese and non-obese individuals. Within the same population, subjects with an enlarged waist circumference had higher SAF than subjects without an enlarged waist circumference. These findings imply that not general obesity but an enlarged waist circumference, or visceral obesity has more impact on SAF within individuals with MetS.

Next, our results may not be compared directly to those from Monami et al. [34] who have also shown that SAF levels are elevated among diabetic individuals with MetS. Mean SAF was higher than in our study as their cohort primarily consisted of type 2 diabetic individuals with a mean diabetes duration of 12 years. Individuals with type 2 diabetes have in general higher SAF than subjects without diabetes [9]. Furthermore, subjects in their study were older and had a higher prevalence of CVD, both are associated with higher SAF [11, 14].

In addition to the observation of higher SAF levels in subjects with MetS, we also found that a higher number of individual MetS components coincides with even higher SAF Z-scores. A previous study has already shown that a higher number of MetS components was associated with higher serum AGEs levels [35]. Clinical studies have demonstrated that a higher number of MetS components is associated with both incident CVD and type 2 diabetes [36–38]. Klein et al. showed that individuals with one MetS components had 2.5% risk of incident CVD in the next 5 years whereas subjects with four or more MetS components had an almost 15% risk. The 5-years risk for type 2 diabetes increased from 1.1% (one MetS component) to 17.9% (≥four MetS components) [36]. An 11-year follow-up study demonstrated an almost linear relationship between the number of individual MetS components and the risk of coronary heart disease in subjects without a history of CVD or type 2 diabetes [38]. Therefore, we suggest that an increase in SAF may reflect an even higher risk of type 2 diabetes and CVD.

It has been reported that the prevalence of both MetS and its components differ between men and women [39]. In men, we observed that elevated blood pressure was the most prevalent MetS component. Moreover, we found that subjects in the highest SAF group had a higher prevalence of elevated blood pressure compared to individuals in the intermediate or lowest SAF group. A few studies have described the association between SAF and elevated blood pressure, but the results are contradictory. For example, in renal transplant recipients, systolic blood pressure was positively associated with SAF [40] whereas neither systolic nor diastolic blood pressure was related to SAF in a recent Japanese study among type 2 diabetic individuals [41]. A recent study by Botros et al. [42] showed that both systolic and diastolic blood pressure were significantly associated with SAF. They showed that individuals with elevated blood pressure had a higher odds for having a SAF level > median, compared to subjects without elevated blood pressure. Elevated blood pressure may well be a consequence of increased AGE accumulation. A recent study demonstrated that carotid-femoral pulse-wave velocity and central pulse pressure were independently associated with plasma AGEs [43]. Several AGEs are able to form crosslinks within collagen in the vascular wall, which may result in impaired vascular elasticity and increased arterial stiffness, causing blood pressure to rise [44, 45]. Our data together with previous studies suggest that AGE accumulation may indeed be involved in the underlying pathophysiology of elevated blood pressure.

In contrast to men among we found elevated blood pressure to be the most prevalent component, we observed that enlarged waist circumference was the most prevalent MetS component among women. Individuals with high SAF levels had a higher presence of enlarged waist circumference compared to subjects with intermediate or low SAF levels. However, in the multivariable analyses, enlarged waist circumference was not significantly associated with SAF. The correlation between waist circumference or BMI and higher SAF levels has been demonstrated among subjects with type 2 diabetes [34]. Recently, it has been shown that SAF levels were elevated among individuals with central obesity compared to lean subjects [33]. Interestingly, Angoorani et al. [46] have demonstrated that dietary consumption of AGEs is associated with MetS, and in particular abdominal obesity. Furthermore, it was observed that an increased consumption of food AGEs was associated with an even higher risk of (abdominal) obesity. They reported that individuals with a high AGEs consumption had a higher fat intake, and indeed it is known that fat contains a significant amount of dietary AGEs per gram of weight [47]. Another possible mechanism that has been reported to increase AGEs accumulation in obese individuals is oxidative stress [48, 49]. Autoxidation of lipoproteins result in the formation of advanced lipoxidation end products formation (ALEs) such as carboxymethyllysine (CML) [1].

Next, we have shown that higher SAF is significantly associated with an increased presence of impaired fasting glucose. After adjusting for HbA1c, impaired fasting glucose was not associated with SAF probably as a consequence of high collinearity. A previous study already demonstrated that among subjects with central obesity, fasting glucose levels were significantly associated with higher SAF in univariate regression analyses but not after adjusting for covariables [33]. Another recent study reported no significant difference in plasma AGEs between women with normal fasting glucose and impaired fasting glucose [50]. As we included in our study subjects without diabetes only, and glucose levels were relatively low (<6.9 mmol/L), this may be the reason why fasting glucose was not associated with SAF. In individuals with diabetes, the formation of AGEs is accelerated particularly due to (chronic) hyperglycaemia [51]. Glucose plays an essential role in the formation of AGEs as protein amino groups and lipids are non-enzymatically glycated to form stable structures on long-lived tissues [4, 52]. A second pathway which leads to the formation of AGEs is through autoxidation of glucose by reactive oxygen species and through formation of carbonyl compounds [1]. It has been shown that AGEs are involved in beta-cell injury, probably caused by inflammation and oxidative stress thought the AGE–RAGE interaction [53]. This may be prevented by consumption of a low-AGE diet, which has been suggested to improve insulin sensitivity [54, 55].

After adjusting for several determinants, having a low HDL cholesterol was significantly associated with higher SAF levels but elevated triglycerides was not. Among individuals with MetS, HDL was just above the limit in men and slightly decreased in women, while statin use was 13% in men and 10% in women. Additional analysis showed that statin users had significantly higher SAF Z scores than those not using statins, also after correcting for age. Triglycerides were slightly elevated in women, but higher in men. Around 1% of individuals with MetS used triglycerides lowering medication. There was no significant difference in SAF Z scores between subject using triglycerides lowering medication and subjects not using this kind of medication. Limited data exist on the association between SAF and both HDL cholesterol and triglycerides. In subjects with type 2 diabetes, HDL cholesterol levels were negatively, and triglycerides were positively associated with SAF [14, 34]. Similar associations have been reported between serum AGEs levels and triglycerides and HDL cholesterol levels in diabetes [56]. In addition, higher consumption of AGE-rich foods was associated with hypertriglyceridemia [46]. HDL cholesterol has been reported to inhibit oxidative modification of LDL cholesterol [57]. A study in 200 type 2 diabetic subjects showed that higher anti-oxidative capacity of HDL was associated with lower SAF but not plasma HDL cholesterol levels itself [58]. Circulating AGEs may impair the capacity of HDL to protect against oxidation of LDL cholesterol which potentially increases oxidative stress. In turn this might accelerate the formation of AGEs [59] and plays a role in atherosclerosis [60].

The strength of this study is its large and well-characterized study population, including high quality data on anthropometric and clinical measurements. This resulted in a good statistical power and the ability to perform stratified analysis. Moreover, this is the largest study in subjects without diabetes and impaired renal failure showing the association between SAF and several cardiometabolic risk factors. A limitation of the study includes the cross-sectional design, which does not allow us to draw any conclusions about causality in the association between SAF levels and the risk of cardiometabolic diseases.

## Conclusion

The present findings of elevated SAF levels in subjects with MetS, the positive association between the number of individual MetS components and higher SAF levels, as well as the observation that higher SAF levels are associated with higher prevalence of the individual components, provide further evidence that accumulation of AGEs may contribute to the pathophysiology of several cardiometabolic risk factors. Prospective studies are needed to demonstrate whether SAF measurement can be used as an additional non-invasive screening tool to detect individuals at high-risk for both CVD and incident type 2 diabetes.

## Abbreviations

AGEs: advanced glycation end products
AU: arbitrary units
BMI: body mass index
CVD: cardiovascular disease
HDL: high density lipoprotein
MetS: metabolic syndrome
SAF: skin autofluorescence
